# *De novo* Transcriptome Assembly and SNP Discovery in the Wing Polymorphic Salt Marsh Beetle *Pogonus chalceus* (Coleoptera, Carabidae)

**DOI:** 10.1371/journal.pone.0042605

**Published:** 2012-08-01

**Authors:** Steven M. Van Belleghem, Dick Roelofs, Jeroen Van Houdt, Frederik Hendrickx

**Affiliations:** 1 Terrestrial Ecology Unit, Biology Department, Ghent University, Gent, Belgium; 2 Department Entomology, Royal Belgian Institute of Natural Sciences, Brussel, Belgium; 3 Department of Ecological Science, VU University Amsterdam, Amsterdam, The Netherlands; 4 Laboratory of Cytogenetics and Genome Research, Leuven, Belgium; Auburn University, United States of America

## Abstract

**Background:**

The salt marsh beetle *Pogonus chalceus* represents a unique opportunity to understand and study the origin and evolution of dispersal polymorphisms as remarkable inter-population divergence in dispersal related traits (e.g. wing development, body size and metabolism) has been shown to persist in face of strong homogenizing gene flow. Sequencing and assembling the transcriptome of *P. chalceus* is a first step in developing large scale genetic information that will allow us to further study the recurrent phenotypic evolution in dispersal traits in these natural populations.

**Methodology/Results:**

We used the Illumina HiSeq2000 to sequence 37 Gbases of the transcriptome and performed *de novo* transcriptome assembly with the Trinity short read assembler. This resulted in 65,766 contigs, clustering into 39,393 unique transcripts (unigenes). A subset of 12,987 show similarity (BLAST) to known proteins in the NCBI database and 7,589 are assigned Gene Ontology (GO). Using homology searches we identified all reported genes involved in wing development, juvenile- and ecdysteroid hormone pathways in *Tribolium castaneum*. About half (56.7%) of the unique assembled genes are shared among three life stages (third-instar larva, pupa, and imago). We identified 38,141 single nucleotide polymorphisms (SNPs) in these unigenes. Of these SNPs, 26,823 (70.3%) were found in a predicted open reading frame (ORF) and 6,998 (18.3%) were nonsynonymous.

**Conclusions:**

The assembled transcriptome and SNP data are essential genomic resources for further study of the developmental pathways, genetic mechanisms and metabolic consequences of adaptive divergence in dispersal power in natural populations.

## Introduction

A vast number of insect species are characterized by remarkable and often discontinuous morphological variation in traits related to dispersal capacity [1], [2]. As variation in such traits determines the ability of populations and species to persist in both patchy and changing landscapes [3], [4], [5], [6], research on the ultimate and proximate causes of dispersal is a central theme in both evolutionary ecology and conservation biology [7], [8]. Theoretical and empirical research on the ultimate cause of dispersal demonstrated that such dispersal polymorphisms are the result of disruptive selection in heterogeneous landscapes in response to habitat persistence [5], [9], [10] and fitness homogenization under spatiotemporal population fluctuations [11], [12], [13], [14](Hendrickx *et al.* under rev.).

Still, only little is known about the molecular basis of this profound phenotypic variation. For instance, it is unclear whether (i) divergence in dispersal traits is caused by a small set genes that exert large effects or by many genes with moderate to small effect, and in which order they are involved in adaptive differentiation [15], [16], [17], (ii) whether adaptations and the evolution of distinct dispersal phenotypes are mainly the result of mutations in coding regions of the genome or rather due to differences in gene expression (i.e. regulatory changes) [18], [19], [20], [21], (iii) if the recurrent appearance of this trait is caused by independent mutations or rather by introgression of standing genetic variation [22], [23] or the release of cryptic genetic variation by changes in epistatic interactions [24], [25], and (iv) how disruptive selection in dispersal traits affects metabolic pathways resulting in genetically correlated changes in other life history traits [26]. Such information is particularly crucial to link the proximate and ultimate mechanisms underlying the recurrent intra- and interspecific evolution of dispersal phenotypes.

The endangered halobiontic ground beetle *Pogonus chalceus* (Marsham, 1802) is a most suitable system to study the molecular mechanisms behind adaptive divergence in dispersal traits. The species exhibits a clear wing polymorphism with both short-winged individuals (brachypterous), long-winged individuals (macropterous), as well as intermediate forms [27]. These differences in dispersal power have been shown to be related to differences in habitat stability and persistence, with long winged individuals occurring primarily in unstable and relatively recent salt marsh areas. The determination of wing size in this species is polygenic as crosses between brachy- and macropterous populations result in the production of individuals with intermediate wing sizes [28]. Divergent selection on wing size likely results in simultaneous selection in other life history traits, as suggested by a strong correlation among populations between average wing size and frequencies of the metabolic enzyme isoforms of the isocitrate dehydrogenase 2 (IDH2) protein [6], [29]. Moreover, within a salt marsh situated at the Atlantic coast in the Guérande region in France, individuals of *P. chalceus* occur chiefly in two habitat types interlaced at a very small scale, i.e. ponds and canals [29]. Salt extraction ponds are mostly occupied by long winged individuals with larger body size and the IDH2-B allozyme. The borders of tidal canals that lead sea water to these ponds are occupied by smaller short winged individuals with the IDH2-D allozyme. While signals of strong divergent natural selection are observed between the ecotypes for the IDH2 allozymes, dispersal power and body size, no differentiation could be detected for neutral markers, suggesting high levels of gene flow among both ecotypes [6], [29], [30]. These findings and the incipient stage of divergence make the salt marsh beetle *P. chalceus* attractive for genetic studies of selection, adaptation, and gene flow.

It has been shown that portions of the wing development gene network are largely conserved among holometabolous insect orders [31], [32]. A number of genes involved in the patterning, growth and differentiation of the wing in *Drosophila* have been identified [33] and characterized in *T. castaneum* [34]. Furthermore, genes involved in the juvenile hormone (JH) and ecdysteroid (ECD) pathway have also been shown to be relevant for the study of insect polymorphisms, including wing polymorphisms [35], [36], [37], [38], [39]. However, little genomic resources are available to study the genetic architecture of dispersal polymorphisms in natural populations of ground beetles, in which intraspecific dispersal polymorphisms can be found abundantly [40], [41], [42]. Considering ground beetles (Carabidae), NCBI reports 306 ESTs from a study comparing seven coleopteran species [43] and a mitochondrial genome of a *Calosoma* species [44]. Other genomic resources comprise mostly single bar-coding gene sequences, such as cytochrome oxidase and ribosomal RNA, used for phylogenetic studies. The only coleopteran species for which the genome has been sequenced is the red flour beetle *Tribolium castaneum* [34], belonging to the Polyphaga suborder. The evolutionary distance of this suborder to the Adephaga suborder, comprising Carabidae species, is estimated to be more than 200 Ma [45].

Short read *de novo* transcriptome analysis has proven to be a valuable first step to study genetic characteristics and allowed researchers to obtain sequence information and expression levels of genes involved in developmental and metabolic pathways, insecticide resistance, candidate transcripts for diapauses preparation based on homology with related organisms and to discover single nucleotide polymorphism (SNP) in all kinds of model and non-model organisms [46], [47], [48], [49].

In this study, we used Illumina short read sequencing for *de novo* transcriptome assembly and analysis of the salt marsh beetle *P. chalceus*. We constructed three libraries covering three life stages, one third-instar larva, one pupa and one adult male beetle. We matched these sequences in a BLAST search to known proteins of the NCBI database and aligned the sequences to the genome of *T. castaneum*. Matches include a number of genes relevant to the study of wing development and dispersal polymorphism. Furthermore, we screened the transcriptome for both conservative SNPs and SNPs resulting in amino acid changes, which will allow genome wide screening of variation between different ecotypes. The resulting assembled and annotated transcriptome sequences constitute comprehensive genomic resources, available for further studies and may provide a fast approach for identifying genes involved in developmental pathways (i.e. wing development, JH, and ECD) relevant to adaptive divergence in this species.

## Materials and Methods

### Tissue material and nucleic acid isolation

The geographical distribution of *P. chalceus* extends along the Atlantic coasts from Denmark up to and including the major part of the Mediterranean coasts [50]. Beetles were captured in the Guérande region, France. No specific permits were required for the described field study. Eggs were obtained from the canal ecotype (short-winged) and raised in a common environment. A larva (third-instar), pupa and imago (male) resulting from the same mother were frozen in liquid nitrogen and subsequently used for sequencing. The sex determination is probably of the XY type [51].

Total RNA was isolated from a complete larva (third-instar), pupa and newly emerged male imago. RNA was extracted using the SV Total RNA isolation System (Promega, Madison, USA) according to manufacturer's instructions and genomic DNA was removed by on-column digest with DNase I. RNA was quantified by measuring the absorbance at 260 nm using a NanoDrop spectrophotometer (Thermo Fisher Scientific, Inc.). The purity of the RNA samples was assessed at an absorbance ratio of OD_260/280_ and OD_260/230_ and the integrity was confirmed on an Agilent 2100 Bioanalyzer (Agilent Technologies, Inc.).

### Illumina paired-end cDNA library construction and sequencing

The cDNA libraries were constructed for the larva, pupa and imago using the TruSeq™ RNA Sample Preparation Kit (Illumina, Inc.) according to the manufacturer's instructions. Poly-A containing mRNA was purified from 2 µg of total RNA using oligo(dT) magnetic beads and fragmented into 200–500 bp pieces using divalent cations at 94°C for 5 min. The cleaved RNA fragments were copied into first strand cDNA using SuperScript II reverse transcriptase (Life Technologies, Inc.) and random primers. After second strand cDNA synthesis, fragments were end repaired, a-tailed and indexed adapters were ligated. The products were purified and enriched with PCR to create the final cDNA library. The tagged cDNA libraries were pooled in equal ratios and used for 2×100 bp paired-end sequencing on a single lane of the Illumina HiSeq2000 (Genomics Core, UZ Leuven, Belgium). After sequencing, the samples were demultiplexed and the indexed adapter sequences were trimmed using the CASAVA v1.8.2 software (Illumina, Inc.).

### De novo transcriptome assembly

The transcriptome reads were *de novo* assembled using Trinity (release 20111126) [52] on the STEVIN Supercomputer Infrastructure at Ghent University (48 cores, 350 G of memory). The three samples (i.e. larva, pupa, and imago) were assembled and analyzed as a pooled dataset. As the Trinity assembler discards low coverage *k*-mers, no quality trimming of the reads was performed prior to the assembly. Trinity was run on the paired-end sequences with the fixed default *k*-mer size of 25, minimum contig length of 200, paired fragment length of 500, 12 CPUs, and a butterfly HeapSpace of 25G (i.e. allocated memory). Prior to submission of the data to the Transcriptome Shotgun Assembly Sequence Database (TSA), assembled transcripts were blasted to NCBI's UniVec database (http://www.ncbi.nlm.nih.gov/VecScreen/UniVec.html) to identify segments with adapter contamination and trimmed when significant hits were found. This adapter contamination may result from sequencing into the 3′ ligated adapter of small fragments (<100 bp). Human and bacterial sequence contamination was investigated using the web-based version of DeconSeq [53], with a query coverage and sequence identity threshold of 90%.

### Functional annotation

The assembled transcripts were subjected to similarity search against NCBI's non-redundant (nr) database using the BLASTx algorithm [54], with a cut-off E-value of ≤10^−3^ and a HSP (high-scoring segment pairs) length cut-off of 33. The publicly available platform independent java implementation of the Blast2GO software [55] was used for blasting and to retrieve associated gene ontology (GO) terms describing biological processes, molecular functions, and cellular components [56]. Top 20 blast hits with a cut-off E-value of ≤10^−6^ and similarity cut-off of 55% were considered for GO annotation. Next, to get an idea of the amount of genes of the *T. castaneum* transcriptome are covered by *P. chalceus* transcripts, assembled transcripts were aligned to the Tribolium Official Gene Set [34], [57] using the PROmer pipeline of the MUMmer 3.0 software [58] with default parameters. The presence of open reading frames (ORFs) was investigated using the ORF-predictor server with an ORF cut-off length of 200 bp [59].

### Genes of interest

To guide our search for wing development genes, we used a previously generated list of *Tribolium castaneum* (Table S13b Richards *et al.* 2008 [34]). To find *P. chalceus* wing development orthologs, we used *T. castaneum* protein sequences in a local BLAST search (tBLASTn) querying the assembled *P. chalceus* transcriptome sequences. Hits with an E-value less than 1e-15 were examined. The most significant hit was considered to be the putative *P. chalceus* orthologue of the wing development gene in *T. castaneum*. Subsequently, the *P. chalceus* transcript sequence was used in a reciprocal blast to the NCBI nr database. If the BLAST and reciprocal BLAST matched, we assigned orthology to that sequence. For the *apterous* gene, we extracted sequences of *D. melanogaster*, *T. castaneum*, *A. mellifera* and *A.pisum* from GenBank and constructed a neighbor-joining tree of the protein sequences with MEGA 5.0 [60], bootstrapped 1000 times. The methodology used is similar to that of Brisson *et al.* 2010 [61].

Next, genes involved in the juvenile hormone (JH) [62] and ecdysteroid (ECD) [63] pathway in *T. castaneum* were extracted from the KEGG pathway database [64] and the same procedure for orthologue discovery for wing development genes was followed. The assembled transcriptome was also investigated for the presence of the isocitrate dehydrogenase 2 (IDH2) gene, which has been shown to be strongly correlated with dispersal power in *P. chalceus* [6], [29]. For this; the *T. castaneum* protein sequence of the gene homologues to IDH2 (XP_970446) was blasted to the *P. chalceus* transcript.

### Mapping reads to reference transcriptome

To align the reads back to the assembled reference transcriptome the Burrows—Wheeler Aligner (BWA) program [65] and the Bowtie aligner [66] were used. BWA was used for variant analysis. Reads were mapped for each sample (i.e. larva, pupa, and imago) separately to the assembled transcriptome based on the pooled read data. The BWA default values for mapping were used, except for number of threads (-t) = 8 and maximum number of alignments (sampe -n) = 40. Under these settings, read pairs mapping to multiple equally best positions are placed randomly. Properly paired reads with a mapping quality of at least 20 (-q = 20) were extracted from the resulting BAM file using SAMtools [67] for further analyses. Properly paired is defined as both left and right reads mapped in opposite directions on the same transcript at a distance compatible with the expected mean size of the fragments. The high mapping quality ensures reliable (unique) mapping of the reads, which important for variant calling.

As reads can map to multiple genes or isoforms and we have no available reference genome, we used the RSEM software [68] to assign reads to genes and isoforms and to count transcript abundances. RSEM requires gap-free alignments and therefore the Bowtie aligner (older version, not Bowtie 2) was used and properly paired reads were extracted. RSEM and Bowtie were used as implemented in the Trinity software package [52]. Bowtie mapping parameters were set as follows: maximum number of mismatches allowed (-v) = 2, number of valid alignments per read pair (-k) = 40. Setting the –k parameter allows reads to align against up to 40 different locations. The old version of Bowtie does not report mapping quality and, hence, does not enable filtering on this parameter. We compared the three developmental stages for transcript composition. Uniquely expressed genes for each life stage were counted and investigated for Gene Ontology (GO) composition.

### Variant analysis

Only reliable properly paired BWA mapped reads were considered for Single Nucleotide Polymorphism (SNP) calling. Indels were not considered because alternative splicing impedes reliable indel discovery. SNPs were called using the SAMtools software package [67]. Genotype likelihoods were computed using the SAMtools utilities and variable positions in the aligned reads compared to the reference were called with the BCFtools utilities [69]. Using the varFilter command, SNPs were called only for positions with a minimal mapping quality (-Q) and coverage (-d) of 25. The maximum read depth (-D) was set at 200. The reference is based on all three samples combined. Therefore, to compare the variational composition of the samples, we extracted only heterozygous SNP positions (i.e. Max-likelihood estimate of the site allele frequency≈0.5) from each sample for the unigenes. Unique and shared SNPs were extracted with the VCFtools software [70]. SNPs located in an open reading frame (ORF) ≥200 bp were extracted. A custom perl script was used to test whether these SNPs resulted in an amino acid change in the predicted ORF.

## Results and Discussion

### Sequencing, transcriptome assembly and validation

**Figure 1:**
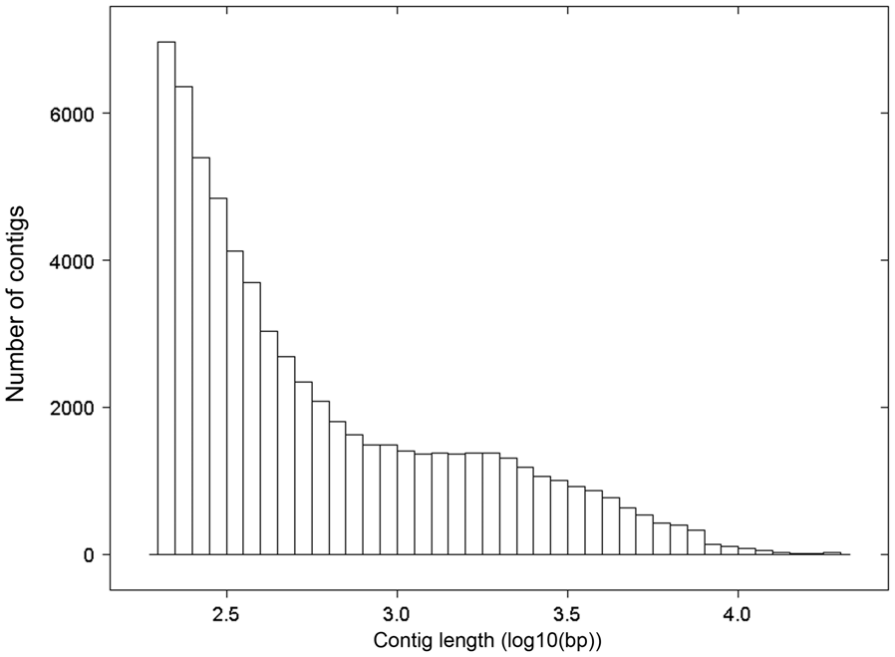
Contig length distribution of Trinity assembly for *Pogonus chalceus*. All assembled contigs were included.

Three developmental stages (one third-instar larva, pupa and male adult beetle) were barcode tagged and sequenced on one lane of an Illumina HiSeq2000 sequencer. Sequencing of cDNA libraries generated a total of 184,749,261 raw paired end reads with a length of 101 bp, resulting in a total of 37.32 giga bases. The raw sequence reads were of good quality (≥20 Phred score). A summary of sequencing, assembly and annotation results for the three samples and the pooled reads dataset is presented in Table 1. For the pupa sample, remarkably less reads were sequenced. Reads were assembled using the RNAseq *de novo* assembler Trinity [52]. The complete read dataset assembled into 65,766 contigs, clustering into 39,393 isoform clusters (i.e. unigenes). We selected the longest transcript as the representative for each cluster. The size of the contigs ranged from 200 (minimum contig length) up to 19,606 bp, with a mean length of 1,046 bp and totaling 68,799,644 bp for all contigs (Figure 1) and a mean length of 869 bp totaling 34,249,556 bp for the unigenes. The top longest (>16,000 bp) assembled sequences were inspected for correctness. Overall these extremely long transcripts matched long gene sequences present in NCBI's nr database, indicating that these sequences are not the result of chimerical assembly errors due to repeat regions in the genes. The longest transcript (19,606 bp) also matches the *D. melanogaster* dumpy gene, a gigantic extracellular protein required to maintain tension at epidermal cuticle attachment sites [71].

**Table 1:** *P. chalceus* transcriptome sequencing, assembly and annotation summary.

Stage		Larva	Pupa	Imago	ALL
Sequencing	Sequencing reads (101 bp paired end)	66,595,267	48,251,298	69,902,696	184,749,261
	Bases (Gbp)	13.45	9.75	14.12	37.32
Assembly	Trinity assembly (Transcripts)				65,766
	Unigenes (Isoform clusters)				39,393
	N50 length (bp) (Unigenes)*				1,904
	Max length (bp) (Transcripts)				19,606
	Max length (bp) (Unigenes)				19,606
	Mean length (bp) (Transcripts)				1,044
	Mean length (bp) (Unigenes)				868
	Median length (bp) (Transcripts)				422
	Median length (bp) (Unigenes)				365
Annotation	Transcripts with BLAST results				29,358
	Unigenes with BLAST results				12,987
	Transcripts annotated with GO terms				17,756
	Unigenes annotated with GO terms				7,589
Mapping	Read mappings (properly paired)	83,539,754	53,814,547	85,597,567	
(BWA)**	Properly paired reads (%)	92.6	90.4	93.1	
	Mean coverage (properly paired)	93.7	55.2	111.6	
	Median coverage (properly paired)	0.93	0.91	2.27	
Mapping	Read mappings	143,056,584	97,896,830	156,747,118	
(Bowtie)**	Properly paired reads (%)	86.8	87.2	87.7	
	Mean coverage (properly paired)	132.98	78.54	150.71	
	Median coverage (properly paired)	1.95	2.21	4.67	

* Contig length for which half of all bases in the assembled sequences are in a sequence equal or longer than this contig length.

** Reads of each sample were mapped to the assembled transcriptome of the pooled data (ALL).

Bacterial and human transcriptome contamination was negligible. Fifty and fifty-seven unigenes were identified by DeconSeq [53] as bacterial and human contaminant sequences, respectively. However, these sequences were short in length (289 bp (SD = 148) and 251 bp (SD = 60) for bacterial and human contaminants, respectively) and most likely represent conserved protein regions.

All sequencing reads were deposited into the Short Read Archive (SRA) of the National Center for Biotechnology Information (NCBI), and can be accessed under the accession number SRA050429. The assembled transcriptome was submitted to the Transcriptome Shotgun Assembly Sequence Database (TSA) and can be accessed through the GenBank accession numbers JU404687–JU470452.

### Functional annotation

**Figure 2:**
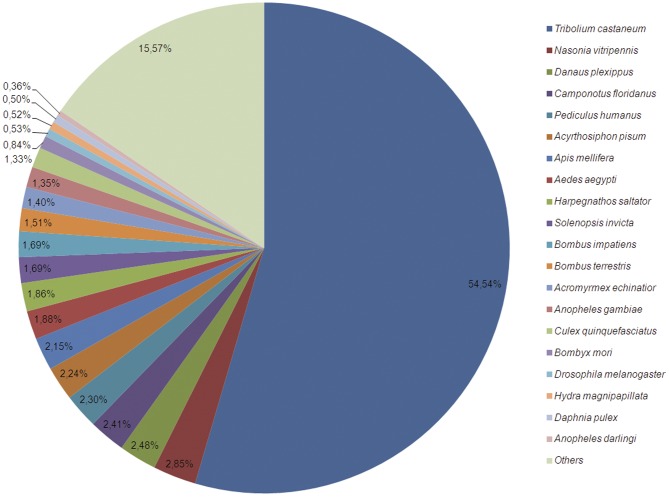
Species distribution of top BLASTx results. The pie chart shows the species distribution of unigenes top BLASTx results against the nr protein database with a cutoff E value<1e^−3^.

**Figure 3:**
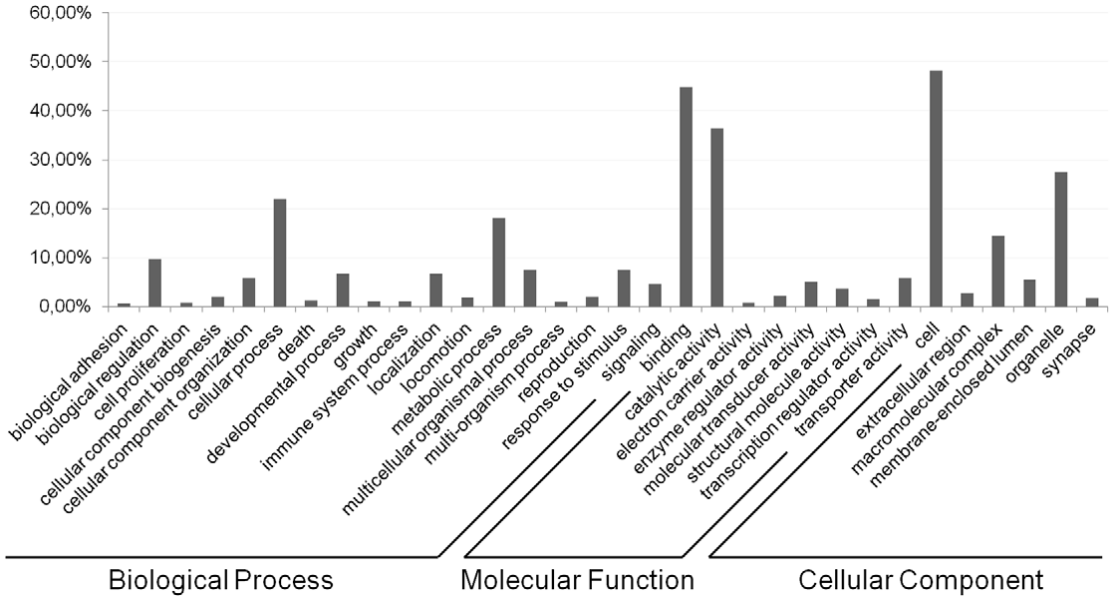
Gene Ontology (GO) categories of the unigenes. Distribution of the GO categories assigned to the *Pogonus chalceus* transcriptome. Unique transcripts (unigenes) were annotated in three categories: cellular components, molecular functions, biological process.

From the assembled unigenes, 12,987 (33.0%) showed significant similarity (E value<1e^−3^) to proteins in NCBI's non-redundant (nr) database, with an average best-hit amino acid identity of 70.5% (SD = 14.2). As expected, the majority of the sequences had top hits to *T. castaneum* proteins (54.5%) (Figure 2), the only Coleoptera species for which a complete genome is available. Other insects resembling *P. chalceus* sequences are divided across different insect orders, the most relevant being Hymenoptera (*Nasonia vitripennis* (2.85%), *Camponotus floridanus* (2.41%), *Apis mellifera* (2.15%), *Harpagnathos saltator* (1.86%)), Lepidoptera (*Danaus plexippus* (2.48%)), Hemiptera (*Acyrthosiphon pisum* (2.24%)), and Diptera (*Aedes aegipty* (1.88%)). The only non-Arthropoda species with top blast hits worth mentioning is *Hydra magnipapillata* (0.53%).In total 7,589 (19.3%) *P. chalceus* unigenes were assigned Gene Ontology (GO) terms based on BLAST matches to sequences with known function. The functional classification based on biological process, molecular function and cellular component is depicted in Figure 3. Among the biological process terms, a significant percentage of genes were assigned to cellular (22.1%) and metabolic (18.0%) processes. Molecular functions were for a high percentage assigned to binding (44.8%) and catalytic activity (36.4%), whereas many genes were assigned to cell part (48.2%) and organelle (27.5%) for the functional class cellular component. These observations are in accordance with observations of metabolic processes in other transcriptomic studies on insects [47], [48], [72], [73], [74].Redundancy is expected in the assembled transcriptome due to the stochastic process of sequencing and the heuristic nature of the assembly process, which can result in the fragmented assembly of genes. To assess how many actual unique genes we have found in our data, we aligned the obtained unigenes to the 16,645 official genes reported for *T. castaneum*. Of these *Tribolium* genes, 6,883 were covered by *P. chalceus* transcripts based on the PROmer alignments [58], with a mean percent similarity of 76.2% (SD = 10.4). Next, mining the alignments shows that 764 of these *Tribolium* gene hits have more than one hit by unique *P. chalceus* transcripts (comprising 1,837 unigenes). For the transcripts with a PROmer alignment to a *Tribolium* gene this corresponds to a maximal redundancy of 15.6% ((1,837-764)/6,883). However, further investigating these multiple hits showed that most comprise genes that belong to the same gene family (i.e. paralogs). Only 272 *Tribolium* genes are matched by multiple non-overlapping *P. chalceus* contigs (comprising 649 unigenes) and align to different portions of the same gene. This reduces the redundancy to 5.5% ((649-272)/6,883). Hence, the contig sets that are different portions of the same gene do inflate the gene counts for *P. chalceus* to only a minor extent.

**Figure 4:**
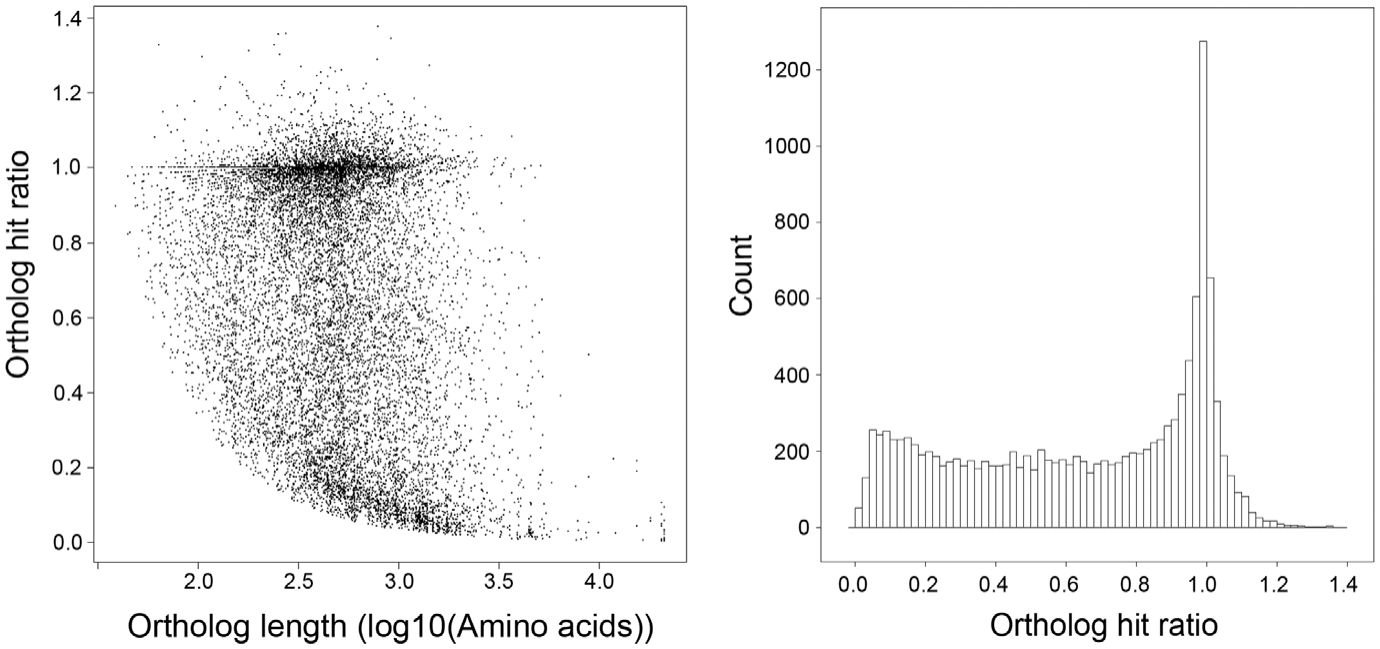
Relationship between ortholog hit ratio and ortholog length (A) and distribution of ortholog hit ratios (B). Ortholog hit ratios were calculated for contigs with BLASTx results. A ratio of 1.0 indicates the gene is likely fully assembled.

We calculated the “ortholog hit ratio” as described in O'Neil *et al.* 2010 [75] by dividing the length of the putative coding region of a unigene by the length of the ortholog found for that unigene. For this, each unigene and its best BLASTx hit were considered orthologs and the hit region in the unigene is considered to be a conservative estimator of the “putative coding region”. In this way, the ortholog hit ratio gives an estimate on the amount of a transcript that is represented by each unigene. Ratios greater than 1.0 can indicate insertions in unigenes. Figure 4(A) shows that the completeness of the assembled transcripts decreases for very long genes. However, for genes with a length <12,000 bp this relationship disappears, which shows that the sequencing design and Trinity assembler succeed well in assembling both short and long transcripts. The distribution of ortholog hit ratios is represented in Figure 4(B). Overall, unigenes with BLASTx results have high ratios, indicating high completeness of these transcripts. Of the 12,987 transcripts with BLASTx results, 4,567 genes have a ratio ≥0.9 and 8,300 have a ratio ≥0.5.

**Figure 5:**
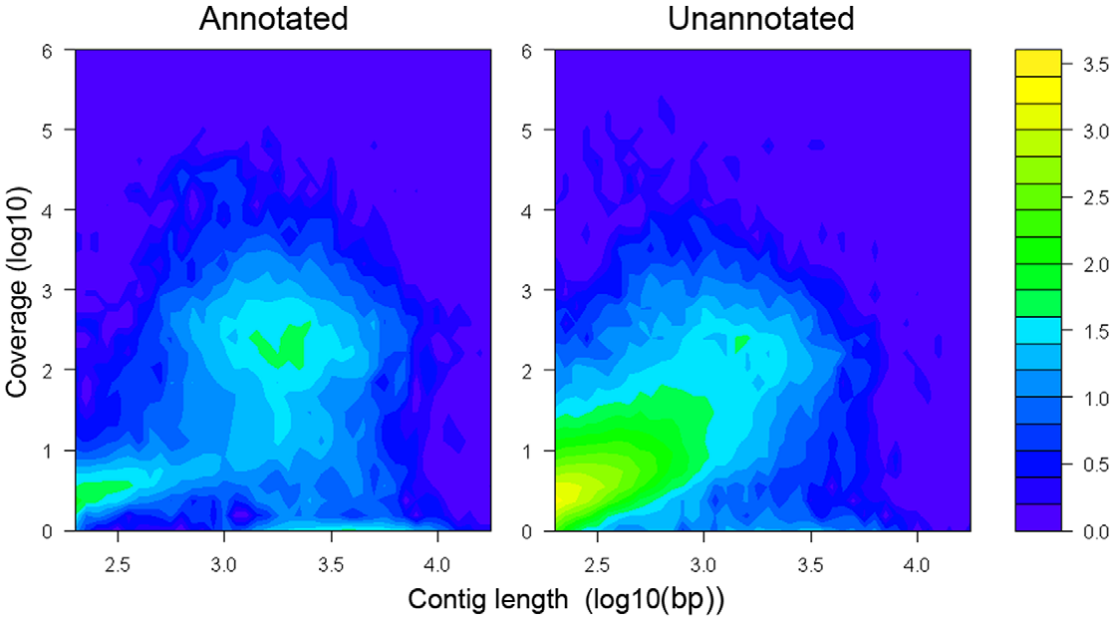
Contour plot of length and coverage distribution of annotated (left) and unannotated (right) unigenes. Transcripts were annotated using Blast2GO. Reads were mapped using BWA. For the annotated transcripts, mean length and coverage was 2,139 and 932, respectively. For the unannotated transcripts, mean length and coverage was 567 and 224, respectively. The color bar shows the log10 transformed count values.

A high percentage of unigenes (31,804; 80.7%) could not be assigned a GO term. Examining the length and coverage distribution of these annotated and unannotated unique transcripts shows that most reads (68.8%) are, however, mapped to annotated transcripts. Furthermore, a major portion of the unannotated transcripts consist of assembled transcripts with very low coverage values and short length (Figure 5). For instance, 23,497 (59.6% of all unigenes) of these unannotated transcripts have a length shorter than 500 bp and only 3.1% of all reads map to these transcripts. These short low coverage transcripts may represent chimeric sequences resulting from assembly errors, fragmented transcripts corresponding to lowly expressed genes, as well as untranslated regions. The remaining 8,427 unannotated sequences are more likely to represent true gene sequences, which may represent novel genes or less conserved genes for which no annotation is found. 15,765 (40.0%) of the unigenes had an ORF (open reading frame) ≥200 bp, with an average length of 1,040 bp and a median length of 659 bp. 7,203 (45.7%) of these unique sequences with ORFs were assigned GO annotations. The remaining sequences with an ORF ≥200 bp that lack annotation results might represent true gene sequences. From the daphnia genome sequence it was discovered that significant genomic regions without assigned open reading frames are actively transcribed [76]. The functional significance of these regions remains to be elucidated, but such transcripts may also be present in the *Pogonus* transcriptome, which cannot be functionally analyzed. Furthermore, high numbers of unannotated contigs are frequently found in other transcriptome sequencing projects [72], [73], [74], [77] and may give some indication of the limitation of inferring the relevant functions of transcripts assembled from sequence data from species with very limited genomic resources or with long evolutionary distances to model species. On the other hand, Trinity succeeds in assembling a reasonable set of annotated genes despite low coverage values (Figure 5).

### Genes of interest

**Figure 6:**
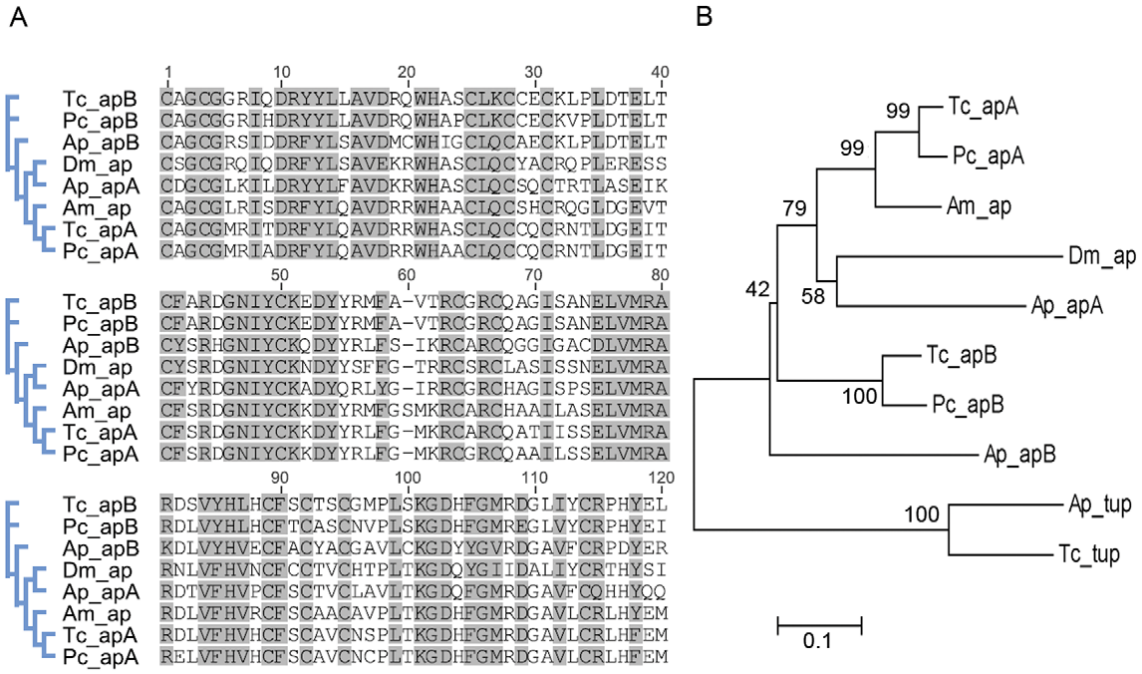
Phylogenetic analysis of the LIM domain of the *apterous* gene. (A) Alignment of protein sequences of the LIM domain region of the *apterous* (*ap*) orthologs and paralogs of *Tribolium castaneum* (Tc), *Acirthosyphon pisum* (Ap), *Drosophila melanogaster* (Dm), *Apis mellifera* (Am) with the presumed paralogs found in the *Pogonus chalceus* (Pc_apA and Pc_apB) transcriptome. (B) Neigbour-joining tree of *ap* protein sequences, rooted with *tailup* (tup). Bootstrap support values are given at each node.

As we are interested in the adaptive divergence of wing length in populations of *P. chalceus*, we began our investigation by searching the assembled transcriptome for orthologous genes known to be involved in wing development in the fruit fly *Drosophila melanogaster*. In particular, we used a previously generated list of the wing development genes reported in the genome of the red flour beetle *Tribolium castaneum* (Table S13b of Richards *et al.* 2008 [34]), which was based on *Drosophila* wing development studies. We found orthologous genes for every wing development gene that we looked for in the assembled *P. chalceus* transcriptome with high confidence (Table 2). Engrailed (en) and invected (inv) blasted to the same *P. chalceus* transcript and reciprocal blast of this component returned engrailed. This is not surprising considering their similarity in sequences and function [78]. Retrieving orthologous genes for the *apterous* (*ap*) gene was problematic as this gene exhibits a duplication in *T. castaneum* and *Acyrthosiphon pisum* [61], [79]. Therefore, we aligned the amino acid sequences of *apterous* genes from *D. melanogaster* (NP_724428), *T. castaneum* (apA: NP_001139341, apB: ACN43342), *Apis mellifera* (XP_392622) and *A. pisum* (apA: XP_001946004, apB: XP_001949543) with those retrieved from BLAST hits to the *P. chalceus* transcriptome (Figure 6). The *apterous* gene is a hox transcription factor and contains two conserved domains; the homeo domain and the LIM-containing region [80]. As we did not retrieve the homeo domain for apB of *P. chalceus*, we only compared the conserved LIM domain region of the *apterous* genes as reported in [61]. To root the tree, we added the closely related LIM-containing gene tailup (tup) of *A. pisum* (XP_001944557) and *T. castaneum* (XP_001815525). The phylogenetic inference indicates that *P. chalceus* exhibits both apterous paralogs that are present in *T. castaneum* and *A. pisum* genome, which were lost in the holometabolous insects *Drosophila* and *Apis*. The relationships are similar as the ones reported by [61].

**Table 2:** List of wing development genes found in *P. chalceus* orthologous to *T. castaneum*.

Function	Gene		Accession *P. chalceus*	Amino acid identity (%)	Ortholog hit ratio
Anterior/Posterior	Engrailed	(en)	Pc_comp5821_c0_seq1	62	1.27
	Invected	(inv)	Pc_comp5821_c0_seq1	56	1.31
	Hedgehog	(hh)	Pc_comp8905_c0_seq1	76	0.96
	Cubitus interruptus	(ci)	Pc_comp4719_c0_seq1	60	1.12
	Patched	(ptc)	Pc_comp7372_c1_seq1	78	0.62
	Decapentaplegic	(dpp)	Pc_comp8429_c0_seq2	64	0.85
	Daughters against	(dad)	Pc_comp5722_c0_seq1	63	1.08
	Brinker	(brk)	Pc_comp8966_c0_seq1	78	0.29
	Optomotor-blind-like	(omb)	Pc_comp6103_c0_seq1	77	0.68
	Spalt-like protein	(sal)	Pc_comp7794_c0_seq1	73	0.87
Dorsal/Ventral	Apterous a	(ap A)	Pc_comp9155_c1_seq1	77	0.76
	Apterous b	(ap B)	Pc_comp10531_c0_seq1	89	0.69
	Notch	(N)	Pc_comp3149_c0_seq1	81	1.02
	Serrate	(Ser)	Pc_comp6451_c0_seq1	80	1.00
	Wingless	(wg)	Pc_comp9580_c0_seq1	96	0.74
	Distal-less	(Dll)	Pc_comp7089_c0_seq1	77	1.08
Vein and sensory	Serum response factor	(srf)	Pc_comp3744_c0_seq2	96	0.36
	Rhomboid	(rho)	Pc_comp9713_c0_seq1	96	0.72
	Knirps	(kni)	Pc_comp8029_c0_seq2	74	0.83
	Knot transcription factor	(knot)	Pc_comp14479_c0_seq1	84	0.61
	Iiroquois	(iro)	Pc_comp4855_c0_seq2	74	1.04
	Abrupt	(ab)	Pc_comp3738_c0_seq3	85	1.00
	Noradrenaline transporter	(net)	Pc_comp9252_c0_seq1	85	0.94
	Delta	(DI)	Pc_comp8811_c0_seq1	70	0.95
	Extramacrochaetae	(emc)	Pc_comp778_c0_seq1	86	1.04
	Achaete-scute	(ASH)	Pc_comp5966_c0_seq1	67	1.09
	Asense	(ase)	Pc_comp12489_c0_seq1	54	1.07
Bodywall/wing	Teashirt	(tsh)	Pc_comp7294_c0_seq1	69	1.13
	Homothorax	(hth)	Pc_comp2739_c0_seq1	87	1.04
	Nubbin	(nub)	Pc_comp7766_c0_seq1	93	0.36
	Ventral vein lacking	(vvl)	Pc_comp4049_c0_seq1	91	1.05
	Vestigial	(vg)	Pc_comp7899_c0_seq1	69	0.74
Hox	Sex combs reduced Scr	(Cx)	Pc_comp5657_c0_seq1	73	1.07
	Prothoraxless	(ptl)	Pc_comp8727_c0_seq1	100	0.31
	Ultrabithorax	(Ubx)	Pc_comp6090_c0_seq1	84	0.97

Subsequently, we performed similar similarity analyses for genes involved in the Juvenile hormone and ecdysteroid pathway. We found orthologous candidates with high certainty for each gene reported in the KEGG insect hormone biosynthesis pathway (Table 3). The length of the ORF of the *P. chalceus* match, compared to the ORF length in *T. castaneum* is also reported.

**Table 3:** List of insect hormone biosynthesis genes.

Function	Gene		NCBI geneID *T. castaneum*	Accession *P. chalceus*	Amino acid identity (%)	Ortholog hit ratio
Juvenile hormone	juvenile-hormone esterase	(JHE)	658208	Pc_comp7235_c0_seq1	62	0.97
	juvenile hormone acid methyltransferase	(JHAMT)	662961	Pc_comp8820_c0_seq1	65	1.01
	juvenile hormone epoxide hydrolase	(JHEH)	659305	Pc_comp841_c0_seq1	74	0.98
	cytochrome P450, family 15	(CYP15A1)	658858	Pc_comp2578_c2_seq2	77	0.95
Molting hormone	ecdysteroid 25-hydroxylase	(PHM)	656884	Pc_comp6141_c0_seq1	72	0.98
(ecdysone)	ecdysteroid 22-hydroxylase	(DIB)	663098	Pc_comp7215_c0_seq2	73	0.70
	ecdysteroid 2-hydroxylase	(SAD)	658665	Pc_comp5946_c0_seq1	64	0.75
	ecdysone 20-monooxygenase	(SHD)	661451	Pc_comp8625_c0_seq2	73	0.69
	cytochrome P450, family 307	(Spo/spok)	658081	Pc_comp9046_c0_seq1	79	0.93
	cytochrome P450, family 18	(CYP18A1)	656794	Pc_comp3811_c0_seq1	86	0.52

Note: Genes were extracted from *T. castaneum* through the KEGG pathway database.

Finally we identified the full coding sequence of the isocitrate dehydrogenase 2 (IDH2) gene (Pc_comp1560_c0_seq1) based on homology to the *T. castaneum* protein sequence (EFA04299; E-value = 0, bit score = 760). The blast result also identified the isocitrate dehydrogenase 1 (IDH1) gene (Pc_comp296_c0_seq1), but with less support (E-value = e-172, bit score = 602).

### Mapping

**Figure 7:**
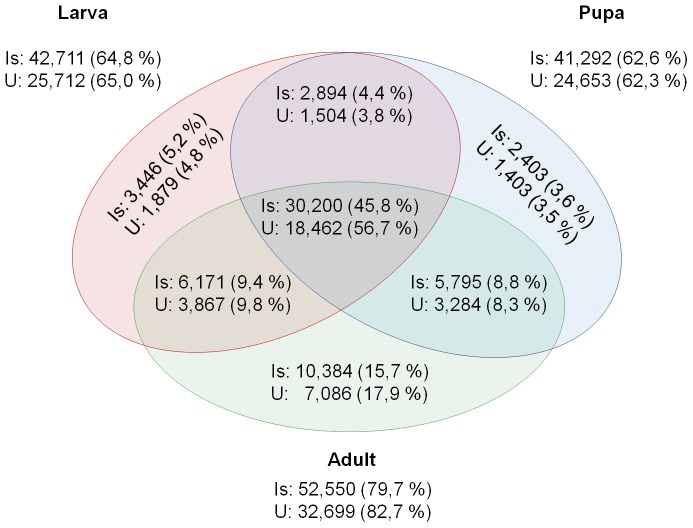
Unique and shared transcript presence of the three developmental stages. The venn diagram shows the unique and shared transcript presence of the three developmental stages (larva, pupa and adult), based on RSEM counts. Reads were assigned to isoforms (Is) or unigenes (U). When RSEM reported a count of at least one, the transcript was reported as present.

Reads for each sample (i.e. larva, pupa, adult) were mapped back to the assembled reference transcriptome based on the pooled data and properly paired reads were extracted (Table 1; Figure 7). Based on the BWA mappings [65], 92.6%, 90.4% and 93.1% of the mapped reads were aligned properly paired when aligning the reads of the larva, pupa and adult sample, respectively, to the assembled reference transcriptome. The mean coverage depth (reads covering each base pair) for the larva, pupa and adult sample is respectively 93.7, 55.2 and 111.6. The Bowtie aligner resulted in a higher mean coverage, owing to reads being mapped to multiple positions. The pupa sample has less mean coverage depth resulting from less sequenced reads.

Some transcripts were represented by many reads. Moreover, 50% of the reads mapped to only 146 transcript sequences and 90% mapped to 2,971 transcripts. Mapping of the reads shows that read coverage is very high. However, the fact that only 149 transcripts consume 50% of all reads may indicate that normalization can be useful for transcriptome assembling. The top twenty of these were investigated and are shown in Table 4. Amongst these transcripts, several are associated with energy metabolism (cytochrome c oxidase subunit II and III, succinate and NADH dehydrogenase and ADP/ATP translocase), locomotion (actin and myosin light chain), transcription (DNA topoisomerase 1) and translation (elongation factor 1 and 2). Ferritin is a protein that stores and buffers iron [81] and its high abundance may resemble an accommodation to high reduced iron concentrations and high oxidative stress in salt marshes [82], [83] or a stress response.

**Table 4:** Top twenty transcripts with most reads assigned.

Accession *P. chalceus*	Nr. reads	Length (bp)	Annotation
Pc_comp0_c1_seq1	21905861	1,272	Unknown
Pc_comp5_c0_seq1	4116337	5,118	Succinate dehydrogenase*
Pc_comp18_c0_seq1	3016196	3,942	Melanization -related protein
Pc_comp23_c1_seq1	2836940	3,453	Unknown
Pc_comp7_c0_seq1	2585095	1,672	Myosin light chain 2**
Pc_comp32_c0_seq1	1912972	3,409	NADH dehydrogenase subunit 4*
Pc_comp4_c3_seq1	1842608	651	Unknown
Pc_comp30_c0_seq1	1823110	8,598	Alpha-tubulin
Pc_comp41_c0_seq1	1788846	1,961	Elongation factor 1-alpha***
Pc_comp1_c0_seq3	1511917	1,714	Actin**
Pc_comp39_c0_seq1	1501260	2,011	Unknown
Pc_comp14_c0_seq1	1505364	6,711	DNA topoisomerase 1***
Pc_comp16_c0_seq1	1501260	2,186	Muscular protein 20
Pc_comp58_c0_seq1	1419825	1,732	ADP/ATP translocase*
Pc_comp13_c0_seq1	1346169	759	Unknown
Pc_comp10_c4_seq1	1217481	1,679	Cytochrome c Oxidase subunit III (coxIII)*
Pc_comp26_c0_seq1	1178489	3,236	Elongation factor 2***
Pc_comp2_c0_seq1	1128159	634	Unknown
Pc_comp19_c1_seq1	1124751	821	Cytochrome c Oxidase subunit II (coxII)*
Pc_comp60_c0_seq1	1114040	2,504	Ferritin subunit

* Associated with mitochondria, energy metabolism and electron transport chain.

** Associated with muscles and movement.

*** Associated with translation or transcription.

### Comparison of the samples

**Figure 8:**
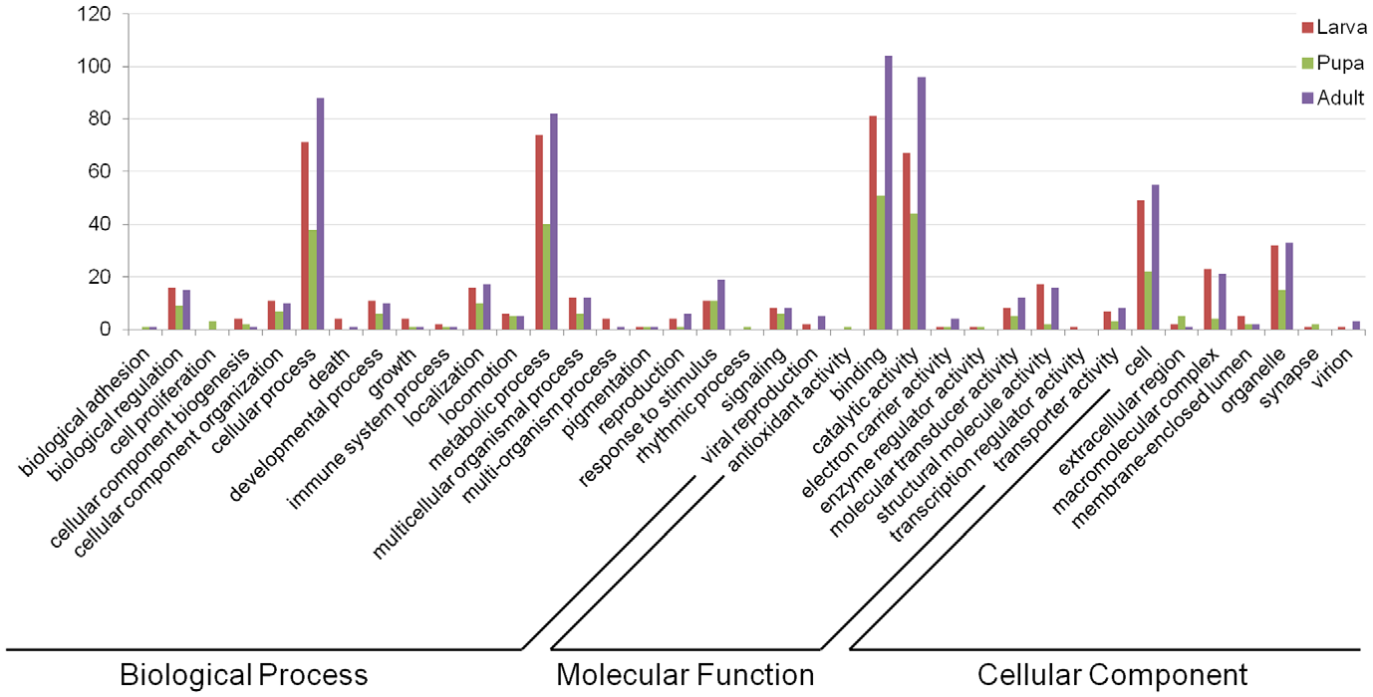
Gene Ontology (GO) distribution assigned to unigenes that are found uniquely in each life stage. Reads were mapped with Bowtie and assigned to genes and isoforms with the RSEM software.

Reads were mapped with Bowtie [66] and assigned to genes and isoforms with the RSEM software [68]. Shared and unique presence of genes and isoforms is shown in Figure 6. 30,200 (45.8%) and 18,462 (56.7%) of the isoforms and unigenes respectively were shared among life stages. 1,879 (4.8%), 1,403 (3.5%) and 7,086 (17.9%) of the unigenes are uniquely expressed in the larva, pupa and adult stage, respectively. Of these uniquely expressed unigenes, only 170, 106, and 243 respectively were assigned GO terms (Figure 8). Overall, the GO term composition of these uniquely expressed transcripts in each life stage corresponds well to the GO term composition of the complete transcriptome. No statistical differences in GO term composition were found between these sets of uniquely expressed genes (FDR<0.1). The higher amount of uniquely expressed genes in the adult stage most likely resulted from more short transcripts being assembled.

### Variant calling

**Figure 9:**
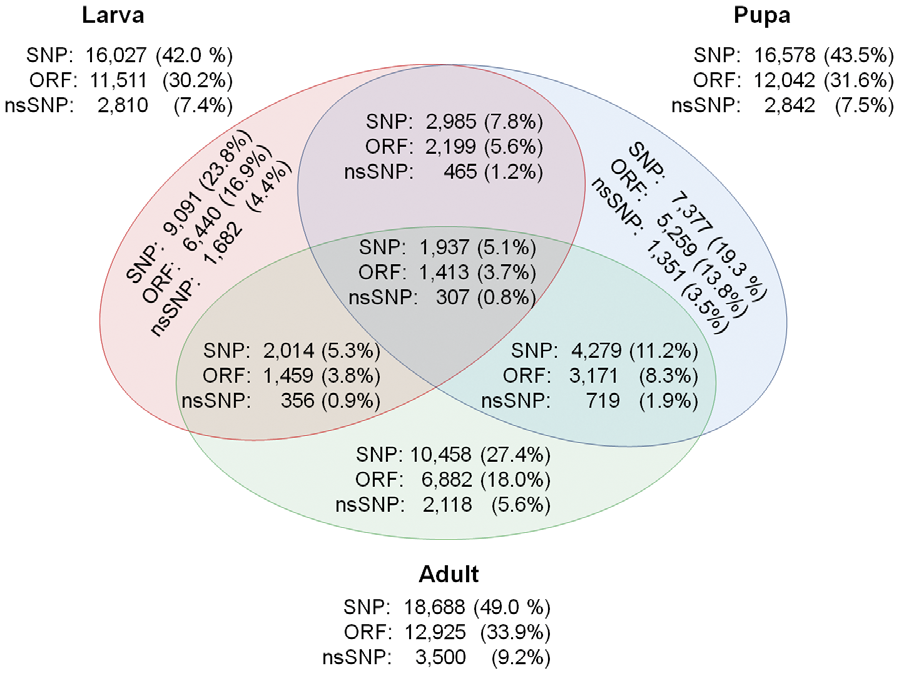
Shared and unique SNPs. Only Heterozygous SNPs are considered from unigenes. The total amount of heterozygous SNPs called in the three samples is 38,141. 70.3% (26,823) of these SNPs were found in an open reading frame (ORF) and 18.3% (6,998) resulted in an amino acid change (nsSNP).

For SNP calling, BWA was used to map the reads of each sample to the reference transcriptome. In total, SAMtools [67] detected 38,141 different heterozygous SNP position in unique transcript sequences using the stringent parameters (i.e. coverage and mapping quality of 25) (Figure 9). This is about one SNP per nine hundred bp of unique transcript sequence (1/898). Of these SNPs, 26,823 (70.3%) were found in a predicted open reading frame (ORF) ≥200 bp and 6,998 (18.3%) resulted in a amino acid change (nonsynonymous SNP (nsSNP)) and are found in 2,907 different unigenes. This results in a percentage of nonsynonymous changes in the coding region of 26.1%, which is lower compared to studies reporting up to 57.3% nsSNPs in coding regions in a single individual of Japanese native cattle [84] and 41 to 47% in human individual resequencing studies [85], [86], but comparable to ratios found in other studies [87], [88].

## Conclusion

In the present study, we sequenced and characterized the transcriptome in the wing polymorphic beetle *P. chalceus*. The assembled sequence data comprising 39,393 unique transcripts provides valuable resources to study wing polymorphism and the adaptive divergence in the face of strong gene flow found in *P. chalceus*. We characterized a large set of genes relevant to wing development and dispersal polymorphism with high significance, including paralogs, giving an indication of the integrity and completeness of the assembled *P. chalceus* transcriptome resulting from short read Illumina sequencing.

We found a high number of putative SNPs (37,492). The combination of SNP calling with ORF prediction allowed us to infer that a large part of the SNPs located in a coding fragment (26,757) result in nonsynonymous nucleotide substitutions (23.2%).

The results show that it is possible to combine transcriptome assembly and characterization with the discovery of both synonymous and nonsynonymous SNPs, providing a framework for further population genomic studies to indentify the molecular basis underlying phenotypic variation of ecologically relevant traits in a non-model species.
